# (Re)thinking the city of proximity for Salutogenic purposes

**DOI:** 10.1093/eurpub/ckac129.078

**Published:** 2022-10-25

**Authors:** A Rebecchi, F Crespi, S Capolongo

**Affiliations:** 1 Department of Architecture, Built Environment, Politecnico di Milano, Milan, Italy; 2 School of Architecture Planning Construction Engineering, Politecnico di Milano, Milan, Italy

## Abstract

As centres of population and human activities, nowadays urban environments are simultaneously the main cause of and solution to a growing number of health-related challenges. In this setting, COVID-19 pandemic has helped reiterate this and serves as a wake-up call and an opportunity to rethink the way we approach cities. Aim of this paper is to research what today seems the most promising urban model for long-term individual and global resilience: the “city of proximity”, namely about inclusive walkable and cycling environments where people can access all basic destinations within reasonable times and distances from home. Therefore, urban proximity dimension, methodological approach and urban features and functions become the main subject of a quanti-qualitative matrix of comparison of five international case studies centred on the topic, by which it is possible to set out general criteria for such model, along with a methodology to measure all cities in its respect. As a result, residential density, functional mix, pedestrian surface, cycle routes, public transport stops, green areas, schools, cultural facilities, sport facilities, retail services and urban gardens make up the fix components of a comprehensive set of 11+n urban features, whose occurrence is investigated through GIS-based analysis within designated distance ranges, creating a comprehensive assessment framework that is adjustable to all urban contexts worldwide. In the end, the application of such framework to the city of Milan finally helps to validate its effectiveness in providing a picture of city-wide accessibility to proximity services, and in highlighting the value of integrated analysis in view of shaping public policies and informed planning choices which put health and sustainability at the centre.

